# Gafchromic EBT3 film provides equivalent dosimetric performance to EBT-XD film for stereotactic radiosurgery dosimetry

**DOI:** 10.1007/s13246-024-01430-z

**Published:** 2024-05-13

**Authors:** Lloyd Smyth, Andrew Alves, Katherine Collins, Sabeena Beveridge

**Affiliations:** Australian Radiation Protection And Nuclear Safety Agency, Australian Clinical Dosimetry Service, Yallambie, VIC Australia

**Keywords:** Audit, Dosimetry, EBT3, EBT-XD, Film, Radiochromic film, Stereotactic radiosurgery

## Abstract

The accurate assessment of film results is highly dependent on the methodology and techniques used to process film. This study aims to compare the performance of EBT3 and EBT-XD film for SRS dosimetry using two different film processing methods. Experiments were performed in a solid water slab and an anthropomorphic head phantom. For each experiment, the net optical density of the film was calculated using two different methods; taking the background (initial) optical density from 1) an unirradiated film from the same film lot as the irradiated film (stock to stock (S-S) method), and 2) a scan of the same piece of film taken prior to irradiation (film to film (F-F) method). EBT3 and EBT-XD performed similarly across the suite of experiments when using the green channel only or with triple channel RGB dosimetry. The dosimetric performance of EBT-XD was improved across all colour channels by using an F-F method, particularly for the blue channel. In contrast, EBT3 performed similarly well regardless of the net optical density method used. Across 21 SRS treatment plans, the average per-pixel agreement between EBT3 and EBT-XD films, normalised to the 20 Gy prescription dose, was within 2% and 4% for the non-target (2—10 Gy) and target (> 10 Gy) regions, respectively, when using the F-F method. At doses relevant to SRS, EBT3 provides comparable dosimetric performance to EBT-XD. In addition, an S-S dosimetry method is suitable for EBT3 while an F-F method should be adopted if using EBT-XD.

## Introduction

The global utilisation of stereotactic radiosurgery (SRS) for intra-cranial indications has increased substantially over the past decade [1, 2] and with it the need for robust and efficient dosimetry protocols. Radiochromic film is an ideal dosimeter for SRS given the demand for a sufficiently high spatial resolution to resolve steep dose-gradients across small target volumes [3]. Typical SRS prescription doses are higher than 20 Gy in a single fraction, often delivered to volumes with a diameter smaller than 2–3 cm.

Gafchromic® EBT3 film (Ashland, Wilmington, DE) has a vendor-recommended upper dose limit of 10 Gy, which limits its use for high-dose clinical applications such as SABR and SRS. This led to the development of the extended dose-range EBT-XD film-type which has an optimal dose range between 0.4 and 40 Gy [4]. Several studies have demonstrated the optical and dosimetric advantages of EBT-XD compared to EBT3, such as: reduced lateral response artifact [4], reduced film orientation effect [5], and increased sensitivity with doses above 5 Gy [3, 5]. The use of EBT-XD for high-dose clinical applications has been validated [3, 6, 7]. However, few studies directly compare the performance of EBT3 against EBT-XD in a clinical context [3].

In addition to the availability of different film-types, there are also varying methods for performing film dosimetry. In general, calibration occurs by fitting a function to the dose versus either pixel value (PV), optical density (OD), or net optical density (netOD). The following definitions have been adapted from AAPM Task Group 235 [8].

The raw pixel value of the query pixel, denoted as PV_q_, ranges from 0 to scanner bit depth (SBD) and the pixel value of a piece of film which has been given zero dose is denoted as PV_0_.

1 $$Optical\,Density \left( {OD} \right) = \log_{10} \left( {\frac{SBD}{{PV_{q} }}} \right)$$

2 $$Net\,Optical\,Density \left( {netOD} \right) = \log_{10} \left( {\frac{{PV_{0} }}{{PV_{q} }}} \right)$$

One choice, with potential significance for both performance and workflow, is the method for determining the netOD for an irradiated film. Specifically, the source of the PV_0_ variable. The netOD formalism has been chosen in this manuscript because this formalism can be used to explicitly identify the source of the PV_0_ value. For establishing PV_0_ the AAPM Task Group 235 suggests using the mean pixel value returned from a region of interest (ROI) of unirradiated film from the same film lot, and with a similar storage history to, the query (irradiated) film [8]. The assumption being that film uniformity is the lesser source of dose uncertainty and that the film scanning and the development conditions (time, temperature, background dose, light exposure) are the greater sources of uncertainty.

There have been few investigations on the influence of film uniformity on film dosimetric uncertainty [5]. The choice of both film-type and dosimetry method has implications for time, cost, and departmental resources, as well as potential impact on the quality of film dosimetry. Given the paucity of available data, the aim of this study was to evaluate the performance of EBT3 versus EBT-XD using different netOD methods, in the context of SRS. Experiments involved benchmarking film-measured doses against traceable standards and SRS treatment plans, as well as making a direct comparison between EBT3 and EBT-XD films.

## Methods

### Study design

Investigations were performed in the context of SRS audits performed by the Australian clinical dosimetry service (ACDS). Three categories of films are defined:

1. Calibration Set: Calibration parameters defined in the next section, irradiated on a Versa HD linear accelerator (Elekta, Stockholm, Sweden) at the Australian Radiation Protection And Nuclear Safety Agency (ARPANSA)*.*
2. Check Films: Irradiated to known doses in a solid water slab either on-site at each audit, using a linear accelerator or GammaKnife system, or at ARPANSA.
3. Audit Films: Irradiated on-site at each audit in a MAX-HD™ anthropomorphic head phantom (IMT, Troy, NY) using treatment plans generated by the department being audited. The prescription dose for the treatment plans was 20 Gy in a single fraction.

Two methods of determining the netOD of a query film were investigated and applied to both the check films and audit films. These methods are defined as 1) stock to stock (S-S), where PV_0_ is determined from an unirradiated film from the same lot as the query film, and 2) film to film (F-F), where PV_0_ is taken from a region of interest (ROI) on a pre-irradiation scan of the query film. All films were therefore scanned prior to irradiation to facilitate the F-F method.

### Films and film processing

Experiments were performed with Gafchromic® EBT3 and EBT-XD film in parallel–both films were irradiated simultaneously. Three different lots per film type were used (EBT3: #06201901, #10171901 and #04022001; EBT-XD: #11271801, #02271901 and #04282001). Films were handled according to the recommendations of AAPM Task Group 235 [8].

A film calibration was performed for each experimental replicate. Each calibration set consisted of 12 films irradiated to known doses (0 to 36 Gy) in a solid water slab (CIRS Plastic Water® DT, Norfolk Virginia, USA) using a 10 × 10 cm^2^ field of 6 MV X-rays, measured at SSD = 90 cm at a depth of 10 cm. An output factor was measured using a Farmer-type PTW 30013 ionization chamber (PTW, Freiburg, Germany) traceable to the Australian primary standards dosimetry laboratory (PSDL) to correct for daily variations in linear accelerator output.

All films were scanned prior to irradiation and then seven days following irradiation as per ACDS protocol. Scanning was conducted with an Epson Expression 12000XL flat-bed scanner (Epson, Suwa, Japan) in transmission mode and at a resolution of 72 dpi. Calibration film sets were captured in a single scan and audit films were scanned with the check films in a single scan capture. Triple channel red–green–blue (RGB) format was used with each pixel having a colour depth of 48 bits (16 bits per channel). No colour corrections were applied. To minimise the impact of spatial heterogeneity in scanner response and lateral response artifact (LRA), films were positioned on the scanner bed using a template. This was to ensure that films were placed on the central axis of the scanner bed and to maintain longitudinal positional consistency between pre- and post-irradiation scans. A glass plate was placed on top of the films to eliminate film curvature during scanning [9]. Images were saved in tagged image file format (tiff).

### Uncertainty

#### Scanner uncertainty

The ACDS performs regular quality control procedures throughout the film process. Intra-scan and inter-scan variability has been measured using high-quality uniform density filters (Neewer, USA). The ACDS maintains an intra-scan variability of less than ± 0.4% and an inter-scan variability of less than ± 0.05%. All of the ROIs examined on the films were contained within ± 2.0 cm of the scanner central axis which had a lateral uncertainty of ± 0.4%. Within the scanning templates used, the error in vertical scanner response was less than ± 0.6% over a distance of 28 cm. Inter-session uncertainty in PV_0_ was ± 0.3% based on quality assurance scans of the filters which are performed every two weeks to monitor scanner performance. We estimate an overall PV uncertainty of ± 0.8% which (based on a nominal calibration curve) gives a dose uncertainty of ± 2.3% at 5 Gy and ± 1.2% at 30 Gy due to the scanner alone.

#### Development uncertainty

In all instances the calibration irradiation was performed on the same day as the check films and audit films. A delay of 7 days before scanning meant that any uncertainty in the darkening is correlated for all films and thus, can be ruled out as a source of experimental uncertainty in this study.

#### Uncertainty due to storage

Calibration films were held at ARPANSA and check and audit films were taken to various audit sites. For a 5 Gy irradiation, a difference in background radiation between calibration and check films needed to exceed 0.005 Gy to contribute > 0.1% uncertainty. One unirradiated control film accompanied the check and audits films for quality control purposes. The control dose measured was negligible. Calibration films, check films and audit films all had similar exposure to incidental light (< 5 min total) during preparation for irradiation and scanning. Otherwise, films were stored in a dark location. Relative darkening (between calibration and check films) due to light exposure is considered negligible.

#### Uncertainty due to film uniformity

Possible causes of variability in film OD are: (1) surface contamination, (2) substate variability and (3) active layer variability. We began this study unable to assign dosimetric uncertainty to each item. An attempt to control for surface contamination has been made by assessing each dosimetric region of interest (ROI) on the calibration and check films. A mean ROI PV was calculated after discrimination of outlier pixels greater than ± 3.0% from the mean, thus removing the contribution from particulates.

Substrate and active layer variability can lead to variability in the OD but measurement of this is coupled with the scanner uncertainty. By examining the difference between using a generic film piece that defines the PV_0_ variable (S-S) or using the pre-scanned query piece that defines the PV_0_ variable (F-F), we can quantify the overall dosimetry uncertainty both with and without film uniformity as a variable.

We assert that the overall uncertainty in dose can be deduced from the experimental results in this study and any variability in dose which is greater than the contribution from the scanner alone shall be attributed to film non-uniformity.

### Calibration

An in-house software program (Daffodil) was developed using Python (version 3.6.5) to convert images of scanned experimental films into corresponding dose-maps via a calibration curve. Calibration curves were generated for each colour channel and described the relationship between the netOD and the known calibration dose. Curves were fitted to the data using a third-degree polynomial function. The netOD for the calibration set was determined by taking the mean pixel value per colour channel from a region of interest (ROI approximate dimensions of 40 × 20 pixels) on each calibration film before and after irradiation. The calibration films were always processed using an F-F method.

### Check film analysis

A total of 17 independent sets of 3 or 4 check films, for a total of 54 check films, per film type were irradiated with known doses ranging from 5 to 30 Gy. The dose returned on the film, D_*Film*_, was determined from a 20 × 40 pixel-sized ROI on each check film using both S-S and F-F methods in Daffodil. Prior to check film irradiation a fixed number of monitor units (MUs) was delivered to a Farmer-type PTW 30013 ionization chamber (PTW, Freiburg, Germany) positioned at the film location. Measurements for both check films and the ionization chamber were taken at a depth of 10 cm, 10 × 10 cm^2^ field size, 6 MV beam energy, with the same ACDS solid water slabs used for obtaining the calibration film measurements. All materials (including ion chambers, phantoms, cables, and electrometers) used for measurements during audits were ACDS owned and calibrated traceable to PSDL. The required known dose to be delivered to the check film, D_*Farmer*_, was achieved by scaling the number of MUs delivered according to the Farmer measurement. The agreement between D_*Film*_ and the known dose (D_*Farmer*_) was evaluated via a check film scale factor, defined as:

3 $$Check\,film\,scale\,factor = \frac{{D_{Farmer } }}{{D_{Film} }}$$

This scaling factor was calculated for each colour channel and applied to each of the fitted calibration curves prior to processing the audit films.

The absolute difference from agreement was also determined for each check-film, and defined as:

4 $$Difference\,from\,agreement = { }abs.\left(\frac{{D_{{Farmer{ }}} - { }D_{{Film{ }}} }}{{D_{Film} }}\right)$$

### Audit film analysis

A total of 6 sets of independent audit films (total of 21 individual audit films) per film type were irradiated using SRS plans with a prescription dose of 20 Gy. Target volumes varied in size from 1 to 2.5 cm in diameter. A microDiamond 60019 detector (PTW) was abutted to each audit film slot in the MAX-HD™(IMT) head phantom during irradiation. EBT3 and EBT-XD audit films were sandwiched in the various film slots, therefore enabling them to be irradiated simultaneously.

Dose-maps of each audit film were generated using both S-S and F-F methods in Daffodil following dose-linear-scaling of the calibration curve based on the corresponding set of check films irradiated at that audit. Then, an in-house MATLAB (version 9.8.0 (R2020a), Natick, Massachusetts: The MathWorks Inc.) program (Marigold) was used to align physical 2D features of the film to the position of the corresponding film-slot visible on the CT dataset for subsequent analysis. Audit films were analysed in three different ways.

Firstly, an ROI dose on the audit film was compared to the dose measured by a coincident microDiamond detector. The agreement between the ROI dose on the audit film and the microDiamond dose was evaluated via a microDiamond scale factor, defined as:

5 $$microDiamond\,scale\,factor = \frac{{D_{Diamond} }}{{D_{Film} }}$$

where D_*Diamond*_ is the dose measured by the microDiamond detector, traceable to the Australian primary standard, and D_*Film*_ is the dose measured on the film at the corresponding location. The absolute difference from agreement was also evaluated as described in Eq. (5):

6 $$Difference\,from\,agreement = abs.\left(\frac{{D_{Diamond } - D_{Film } }}{{D_{Film} }}\right)$$

Secondly, dose-maps from the sandwiched EBT-3 and EBT-XD films were compared to the corresponding dose plane from the DICOM RT plan dose cube and evaluated to using two sets of gamma criteria: 5% dose difference and 1 mm distance agreement (DTA), and 3% dose difference and 3 mm DTA. Percentage dose difference was normalised globally to the prescription dose and a 10% threshold was applied. No positional corrections were applied to account for mis-delivery of the plan during the audit.

Finally, the dose-maps of pairs of sandwiched EBT3 and EBT-XD audit films were aligned using MATLAB for subsequent analysis of per-pixel dose-differences between the film types. The per-pixel dose difference was defined as:

7 $$Per\,pixel\,dose\,difference = \frac{{D_{Film1 } - D_{Film2 } }}{Prescription \,Dose} + 1$$

The average per-pixel difference was determined in two distinct regions; target (> 10 Gy isodose region) and non-target (2 to 10 Gy isodose region).

### Statistics

Analysis of results was performed using Prism (version 9, GraphPad Software, San Diego, CA). The check film and microDiamond scale factors were plotted per colour channel and described using the mean and standard deviation (SD).

Absolute difference from agreement was evaluated per colour channel and the difference between film-type (EBT3 versus EBT-XD for a given netOD method) and netOD method (S-S versus F-F for a given film type) was analysed using Wilcoxon matched-pairs signed rank tests with a Holm-Sidak correction for multiple tests per colour channel. For the comparison of the absolute difference between check-films receiving 10 Gy or less versus more than 10 Gy, a Mann-Witney test was performed. A corrected *P*-value less than 0.05 was considered statistically significant.

## Results

### Calibration curves

**Fig. 1:**
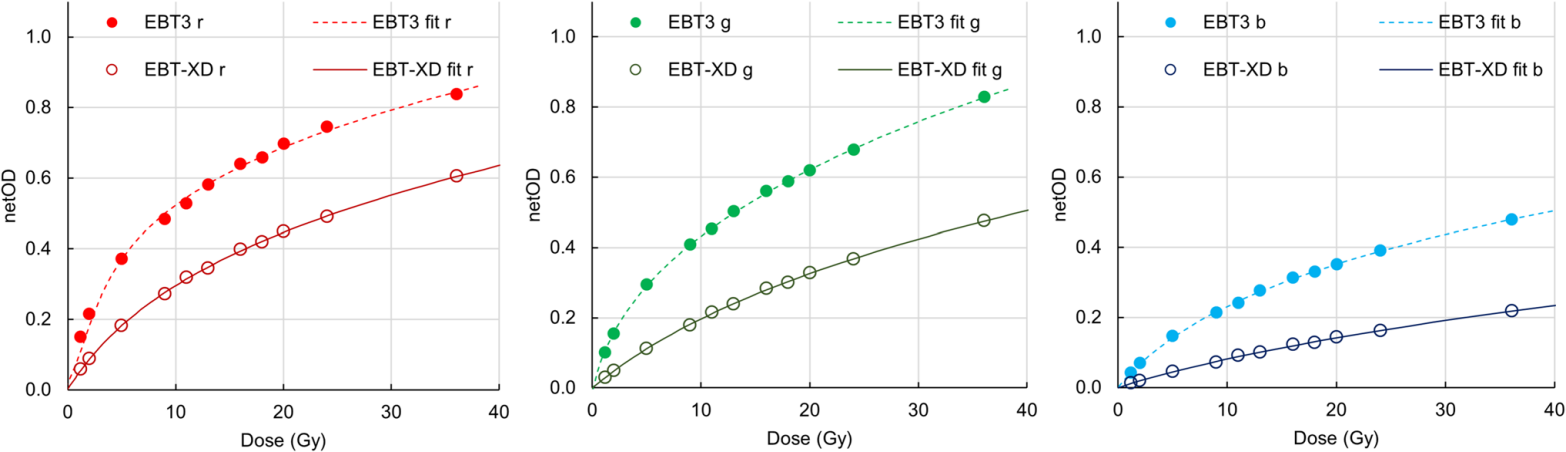
Example calibration curves for the RGB colour channels for an EBT3 and EBT-XD film set

**Fig. 2:**
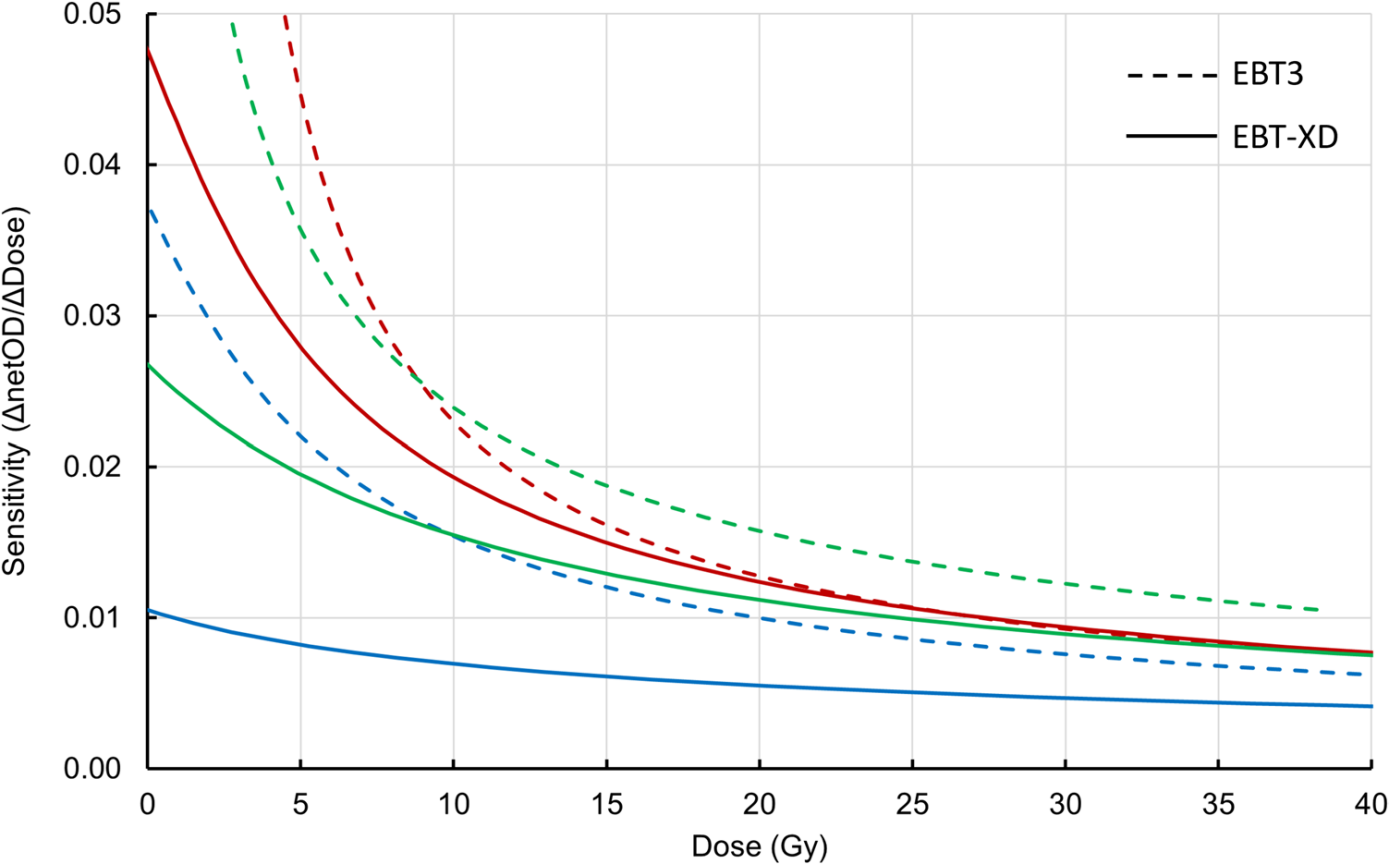
Example sensitivity curves for the RGB colour channels comparing EBT3 and EBT-XD film types. EBT3 is shown as dashed lines and EBT-XD is shown as solid lines, with colours representing the respective channels

Calibration curves (netOD vs dose) are shown with one EBT3 and one EBT-XD film lot from the study as an example in Fig. 1 and the sensitivity (ΔnetOD/ΔDose vs Dose) of these curves is shown in Fig. 2. The response sensitivity of the film for each colour channel is defined as the gradient of the calibration curve at each point [8, 10].

For a fixed uncertainty in PV, a calibration with higher sensitivity will return less dose uncertainty. Examining the sensitivity plots in Fig. 2, we find that EBT3 provides a higher sensitivity calibration up to 30 Gy for green and blue colour channels. The red channel has a lower sensitivity compared to the green channel for doses greater than 9.1 Gy for EBT3 film, whereas the red channel with EBT-XD film is consistently higher in sensitivity than the green channel up to 30 Gy. For the red colour channel at 30 Gy, the sensitivity between EBT3 and EBT-XD is equal. Based on the sensitivity analysis there is no reason to anticipate that EBT-XD would provide a lesser dose uncertainty than EBT3 at doses up to 30 Gy.

### Check-film versus farmer chamber measured dose

**Fig. 3:**
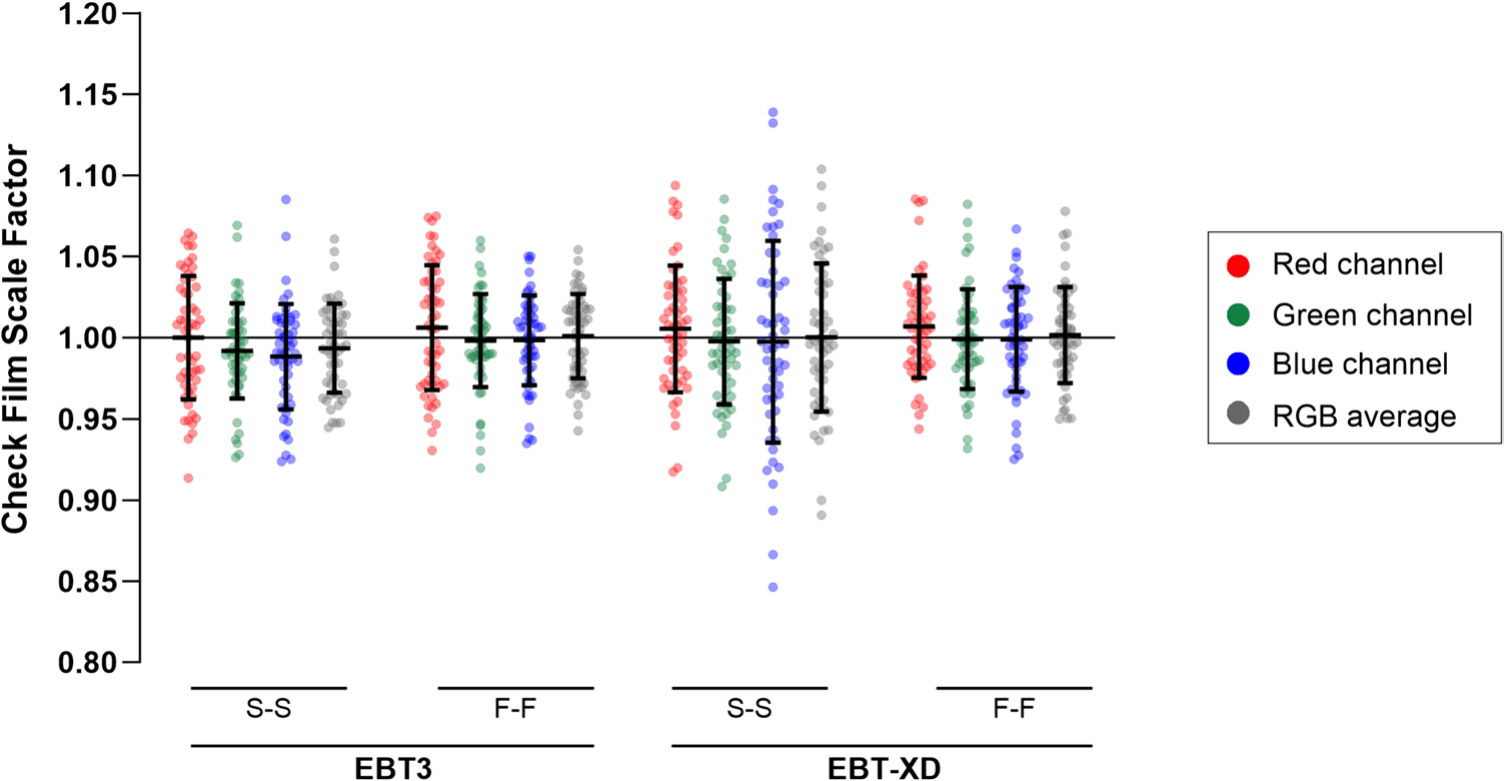
Distribution of check-film scale factors from the check-film analysis (N = 54 pairs of EBT3 and EBT-XD films from N = 17 independent experiments). S-S, stock to stock; F-F, film to film

Check-film scale factors for each combination of film-type and netOD method are shown in Fig. 3 with the corresponding means (of each distribution) and SDs summarised in Table 1.

**Table 1:** Check-film scale factors by channel

Film type	netOD method	Stock-to-Stock				Film-to-film			
	Channel	Red	Green	Blue	RGB	Red	Green	Blue	RGB
EBT3	Mean	1.000	0.992	0.988	0.993	1.006	0.998	0.998	1.001
	SD	0.038	0.029	0.032	0.027	0.038	0.029	0.028	0.026
EBT-XD	Mean	1.005	0.997	0.997	1.000	1.007	0.999	0.999	1.001
	SD	0.039	0.038	0.062	0.046	0.031	0.031	0.032	0.030

The mean of the scale factor distributions for all colour channels, film-types and netOD method combinations were between 0.988 and 1.007 of the Farmer chamber-measured doses.

For EBT3, the red channel had a larger SD in scale factors compared to the green channel for both S-S (± 3.8% versus ± 2.9%) and F-F (± 3.8% versus ± 2.9%) methods, which suggests a higher uncertainty in the red channel for EBT3.

**Fig. 4:**
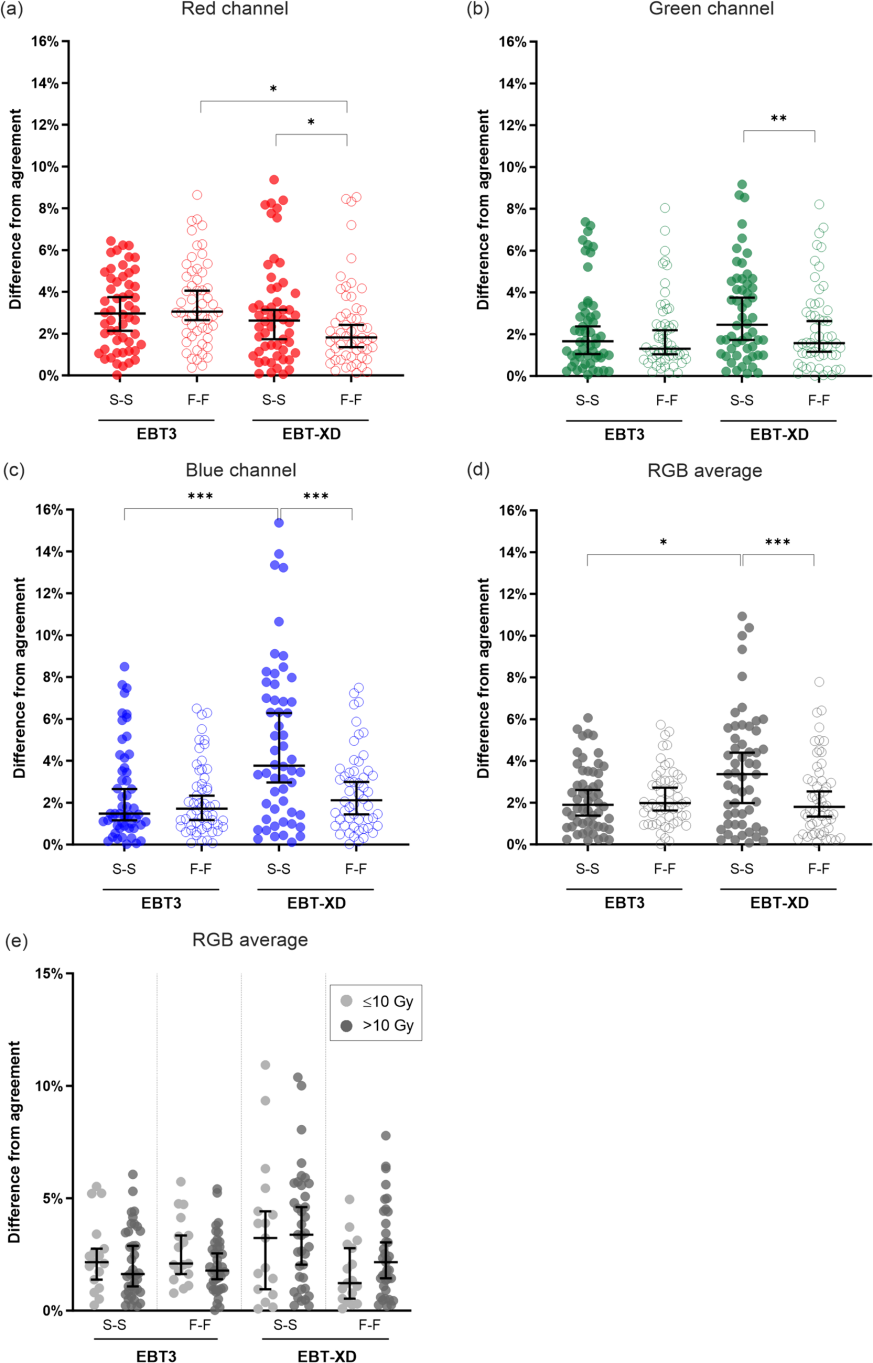
Absolute difference from agreement between film and Farmer measurements of delivered dose. Significant differences between S-S and F-F methods were observed for EBT-XD, but not EBT3, for all channels ((**a**) red channel, adjusted *P* = 0.025; (**b**) green channel, adjusted *P* = 0.001; (**c**) blue channel, adjusted *P* = 0.0004; (**d**) RGB average, adjusted *P* = 0.0004). When using an F-F method, the performance of EBT3 was 1.0% worse compared to EBT-XD (adjusted *P* = 0.025) (**a**). The performance of EBT3 (S-S and F-F) was comparable to EBT-XD for green (**b**) and triple channel dosimetry (**d**). Dose range did not impact EBT3 performance (**e**). * adjusted *P* < 0.05, ** adjusted *P* < 0.01, *** adjusted *P* < 0.001. S-S, stock to stock; F-F, film to film

When examining the absolute difference between film and Farmer measurements (Fig. 4(a)), the red channel was significantly worse for EBT3 compared to EBT-XD when using the F-F method by ± 1.0% (adjusted *P* = 0.025).

The SDs were larger for EBT-XD when using the S-S method compared to both EBT3 S-S and EBT-XD F-F. The blue channel had the largest SD (± 6.2%) in this case (EBT-XD, S-S method), which was reduced substantially when the F-F method was used (± 3.2%). The absolute difference in agreement was significantly improved across all colour channels when using an F-F method for EBT-XD (red channel, adjusted *P* = 0.025; green channel, adjusted *P* = 0.001; blue channel, adjusted *P* = 0.0004; RGB average, adjusted *P* = 0.0004) (Fig. 4(a–d)). These results show that the F-F method has a better uncertainty with dosimetric results for EBT-XD compared to the S-S method.

The dosimetric uncertainty with EBT3 and EBT-XD was comparable when using the green channel only or with the average of the three channels (RGB dosimetry), with the SD of check-film scale factors ranging from ± 2.5 to ± 3.0%. The exception was the S-S method for EBT-XD, where the SDs were ± 3.8% and ± 4.6% for the green channel and RGB average, respectively. Despite the vendor recommended upper dose-limit of 10 Gy for EBT3, there was no significant difference in performance for any film-type or netOD combination when comparing films irradiated to ≤ 10 Gy versus > 10 Gy (Fig. 4(e)).

### Audit film versus microdiamond measured dose

**Fig. 5:**
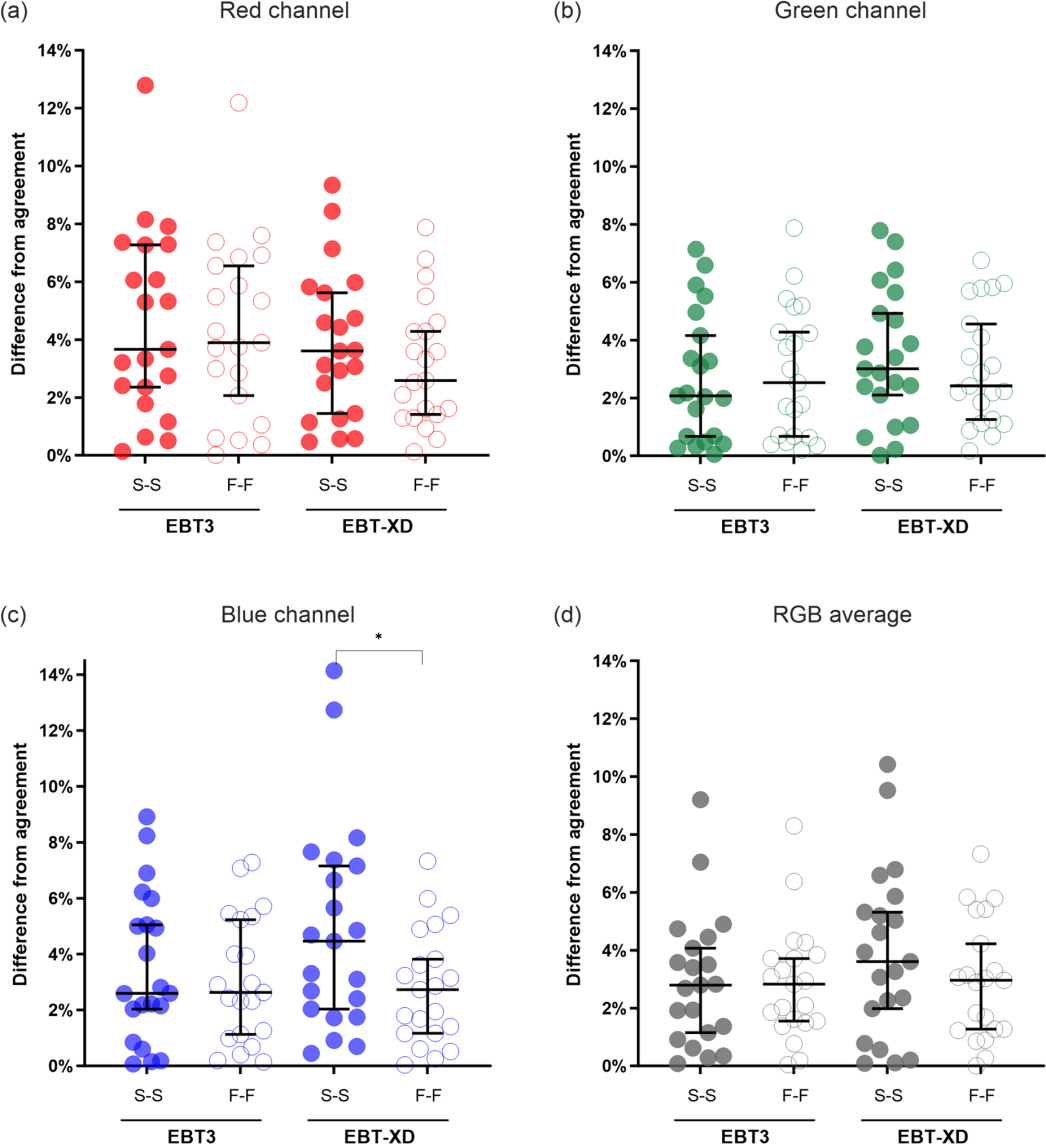
Absolute percentage difference from agreement between microDiamond and film-measured doses in an anthropomorphic head phantom (N = 21 pairs of EBT3 and EBT-XD films, N = 6 audits) for red (**a**), green (**b**) and blue (**c**) channels and for triple channel dosimetry (**d**). A significant difference was observed for the blue channel only when comparing S-S and F-F netOD methods for EBT-XD (adjusted *P* = 0.022). The difference from agreement was comparable for EBT-XD and EBT3 for green and triple channel dosimetry. * adjusted *P* < 0.05. S-S, stock to stock; F-F, film to film

The agreement between audit film and microDiamond measured doses (Fig. 5(a–d)) reflected the check-film versus Farmer-chamber experiment. The absolute difference in agreement was improved for EBT-XD when using the F-F method instead of the S-S method, with significant differences (adjusted *P* = 0.022) observed for the blue channel (Fig. 5(c)). The difference in agreement between EBT3 (S-S and F-F) and EBT-XD (F-F) was comparable, with median differences ranging from 2 to 3% for green channel and triple channel dosimetry.

### Audit film versus planned dose

**Fig. 6:**
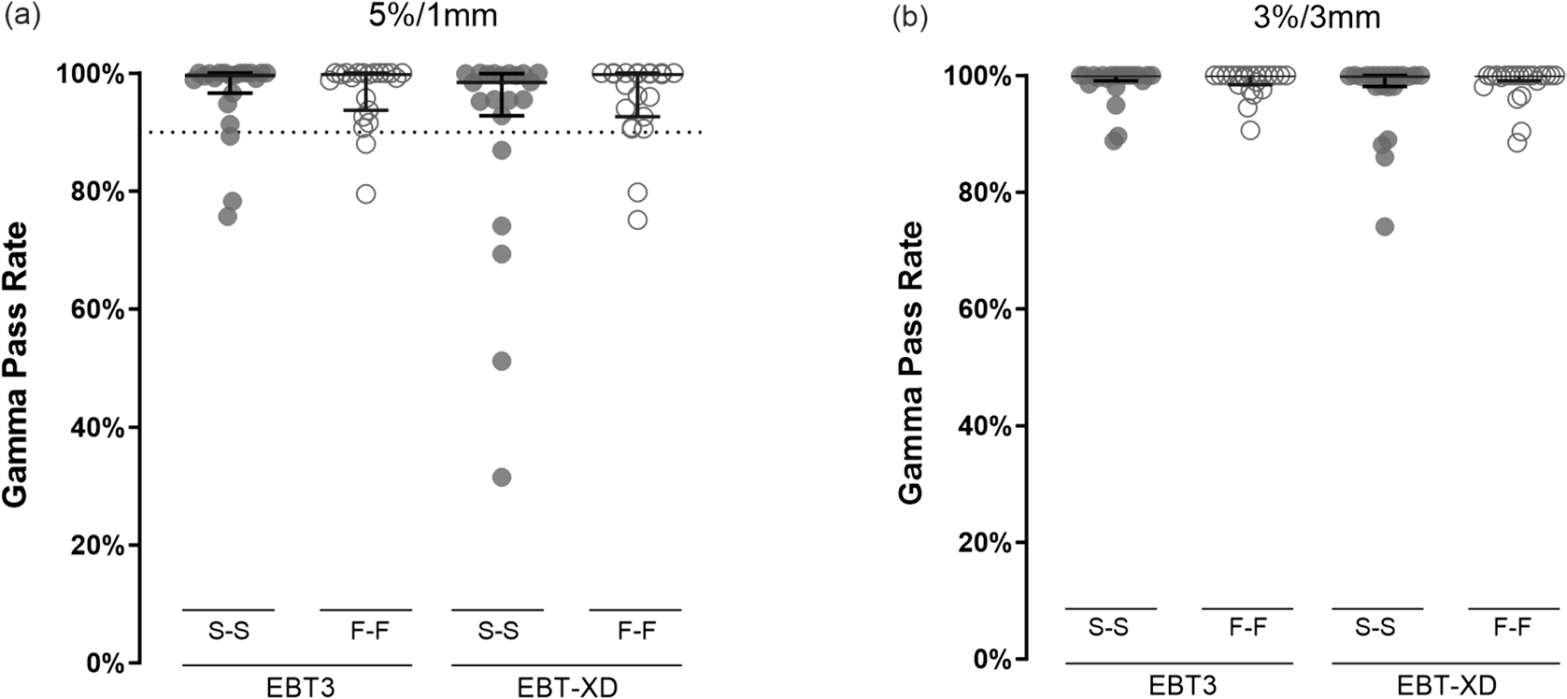
Scoring metrics for audit films (N = 21 pairs of EBT3 and EBT-XD films, N = 6 audits) against the planned dose distribution using gamma criteria and mean distance to agreement. The distribution of results for gamma pass rates for 5%/1 mm (**a**) and 3%/3 mm (**b**) were similar between all film-type and netOD combinations, except for EBT-XD when using an S-S method. The dotted line in panel (**a**) indicates the nominal 90% gamma (5%/1 mm) pass-rate threshold used to score the audit film as passing or out-of-tolerance

Gamma pass-rates for each film-type and netOD combination, using both 5%/1 mm DTA and 3%/3 mm DTA criteria are shown in Fig. 6(a, b), respectively. For the 5%/1 mm criteria, which are used for audit scoring, the median pass-rate for all combinations was greater than 99.5%, except for EBT-XD when using the S-S method, which had a median pass-rate of 98.4% (First quartile to third quartile: 89.9%–99.9%).

### EBT3 versus EBT-XD dose-difference maps

**Fig. 7:**
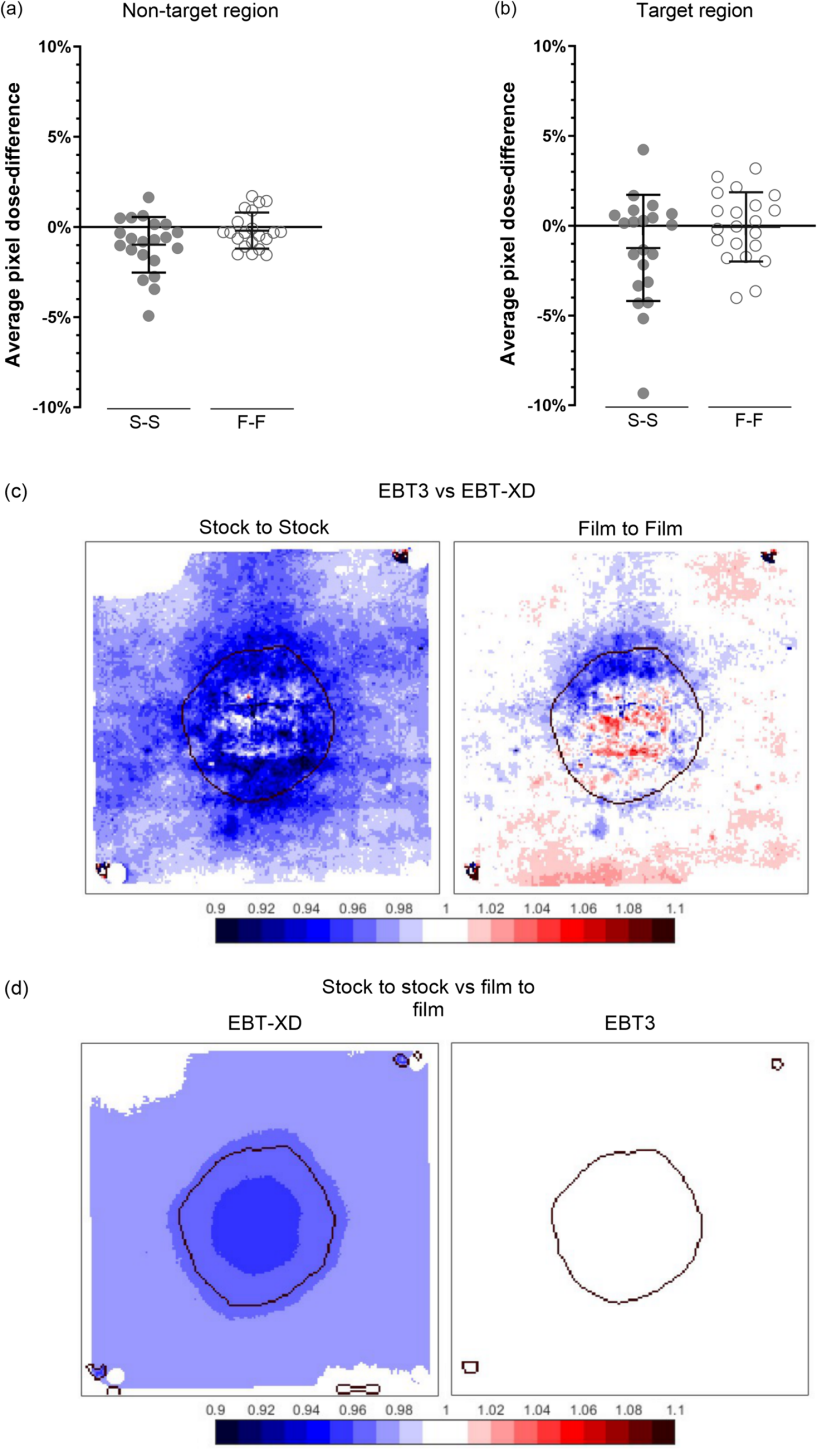
Per pixel dose-difference, normalised to the prescription dose, between sandwiched EBT3 and EBT-XD audit films (N = 21 pairs of EBT3 and EBT-XD films, N = 6 audits) irradiated in an anthropomorphic head phantom. The average normalized per pixel dose-difference between film types was within 2% and 4% of the prescription dose (20 Gy) in non-target (**a**) and target (**b**) regions, respectively, when using the F-F netOD method. Dose-differences between EBT3 and EBT-XD were larger when comparing films using the S-S method, as illustrated by a representative pair of films (**c**). Large dose differences between S-S and F-F methods were more common for EBT-XD, while EBT3 film showed little to no dose-difference between S-S and F-F methods (**d**). Colour bar shows the average per pixel dose-difference, normalized to the prescription dose (20 Gy), as described by Eq. (6). S-S, stock to stock; F-F, film to film

Across all 6 independent audits (N = 21 pairs of EBT3 and EBT-XD audit films), the average per-pixel agreement between sandwiched EBT3 and EBT-XD audit films was within ± 2% and ± 4% of the prescription dose (20 Gy) for the non-target (2—10 Gy) and target (> 10 Gy) regions, respectively, when using the F-F method (Fig. 7(a–b)). The mean ± SD difference between EBT3 and EBT-XD in the target region was 1.2% ± 3.0% and 0% ± 1.9% of the prescription dose for S-S and F-F methods, respectively. In the non-target region, the mean ± SD differences were 1.0% ± 1.5% and 0.2% ± 1.0% for S-S and F-F methods, respectively.

For EBT-XD the mean ± SD per-pixel difference between S-S and F-F methods was − 1.6% ± 1.8% and − 1.0% ± 1.2% of the prescription dose for target and non-target regions, respectively. In contrast, for EBT3, the mean ± SD differences between netOD methods was substantially smaller at − 0.4% ± 0.8% and − 0.2% ± 0.4% for target and non-target regions, respectively. A representative image of EBT3 and EBT-XD dose-difference maps is shown in Fig. 7(c–d), where EBT-XD showed a large variation between S-S and F-F methods.

Compared to audited treatment plans, the median gamma pass-rates for both 5%/1 mm and 3%/3 mm criteria were higher than 99.5% for EBT3 and the distribution of results comparable to EBT-XD. Using a nominal 90% gamma (5%/1 mm) pass-rate as an out-of-tolerance threshold, 20 of 21 audit films had the same audit result based on a comparison of EBT3 (S-S or F-F) and EBT-XD (F-F).

## Discussion

This study involved a direct comparison of EBT3 and EBT-XD film-types using photon doses relevant to SRS while also investigating the influence of film processing method on dosimetric performance. To our knowledge, this is first study to benchmark the performance of EBT3 and EBT-XD film against chamber/detector-measured doses traceable to a primary standard. Benchmarking was performed using reference conditions or SRS treatment plans delivered to an anthropomorphic head phantom.

There were two main findings from this study: (1) EBT-XD has a poorer performance than EBT3 when using the S-S method, and (2) EBT3 was not significantly different to EBT-XD when using triple-channel or green single-channel dosimetry.

While the check-film experiment allowed benchmarking of film performance in highly controlled reference conditions, the audit film results reaffirmed the non-inferiority of EBT3 in the context of SRS treatment plans. It should be noted that microDiamond detectors have inherently higher dosimetric uncertainty compared to Farmer chambers [11], and therefore have reduced utility for benchmarking the performance of film dosimetry. In this study, we observed that the random error in the microDiamond scale factor was greater compared to the check film scale factor (Farmer measurement). We attribute this noise to the uncertainty in microDiamond dosimetry in a modulated treatment plan, and not the film. Therefore, we assess that the microDiamond scale factor results were comparable to the check-film scale factor results. This corroborated both main findings of this study, as stated above.

An additional limitation of this study is that it is not possible to make a distinction between artifacts of the film dosimetry process and errors associated with mis-delivery (geometric or dosimetric) of the audited treatment plans. However, the methodology adopted in this study was aligned with its defined purpose; to make a direct comparison between EBT3 and EBT-XD film types and dosimetry processing methods. Therefore, all EBT3 and EBT-XD films were irradiated in parallel, meaning that any artifact of treatment mis-delivery would apply equally to both film types.

This study also attempted to investigate the film non-uniformity effect by comparing two methods of calculating a netOD (S-S and F-F). It should be noted that only the relative optical transmission of the zero-dose film pieces is used to correct for film non-uniformity in the F-F method. Differences demonstrated between S-S and F-F methods for EBT-XD are not subject to inter-scan variation, given that this comparison was made using the same irradiated film and corresponding scan. Therefore, the possible effect of random variations on the results due to film characteristics cannot be excluded.

The results from this study suggest that the F-F method can partially correct for inter-sheet variation. However, film non-uniformity may also cause relative differences in the response to radiation without measurable differences in transmission at zero dose. The results show that for both film types the F-F method did not return SDs in the predicted 1.2–2.3% range (scanner uncertainty 5–30 Gy). Instead, they were higher for both film types (± 2.6% for EBT3 and ± 3.0% EBT-XD), which suggests the inter-sheet heterogeneity response to radiation remains a detectable source of uncertainty in the F-F method.

EBT-XD had relatively poorer performance when using the S-S method. Statistically significant improvements in the agreement between film- and Farmer-measured doses were observed across all channels when an F-F method was used for EBT-XD, most notably for the blue channel (Fig. 4(c)). A possible explanation for the substantial difference between S-S and F-F methods for EBT-XD is inter-sheet variation within a film lot, given that the blue channel is highly sensitive to the thickness of the active layer of the film [8].

A potential advantage of EBT3 is the ability to use the S-S method, which is less resource intensive than the F-F method as it does not require taking a pre-irradiation scan of each individual film. Taking the background optical density from a piece of film from the same sheet is a potential compromise between the F-F and S-S methods.

## Conclusion

We expected that the F-F method would provide a better uncertainty compared to the S-S method, since background corrections and comparisons are made for each individual piece of film, rather than using a sample average. The results of this study showed a similar range of uncertainties with EBT3 film, but a larger range of uncertainties with EBT-XD. The differences seen between the S-S and F-F methods with EBT-XD film suggest that EBT-XD may not be as homogenous and uniform in response compared to EBT3. This study suggests that an F-F method of processing EBT-XD film may provide better results compared to an S-S method of processing.

## Data Availability

Data sets generated during the current study are available from the corresponding author on reasonable request or through ACDS@arpansa.gov.au. The audit data can be made available in an anonymous capacity, but restrictions apply due to confidentiality, legal, ethical or commercial reasons. Publicly available data can be accessed from https://www.arpansa.gov.au/our-services/testing-and-calibration/calibration/australian-clinical-dosimetry-service.
